# Debridement of Venous Leg Ulcers With Direct-Contact, Low-Frequency Ultrasound: Results of a Randomized, Prospective, Controlled, Clinical Trial

**Published:** 2019-03-13

**Authors:** Oscar M. Alvarez, Martin E. Wendelken, Mark S. Granick

**Affiliations:** ^a^Center for Curative and Palliative Wound Care, Calvary Hospital, Bronx, NY; ^b^Vascular and Wound Care Center, University Hospital, Newark, NJ; ^c^Division of Plastic Surgery, Rutgers New Jersey Medical School, Newark

**Keywords:** debridement, venous ulcers, wound healing, direct-contact, low-frequency ultrasound

**Figure:**
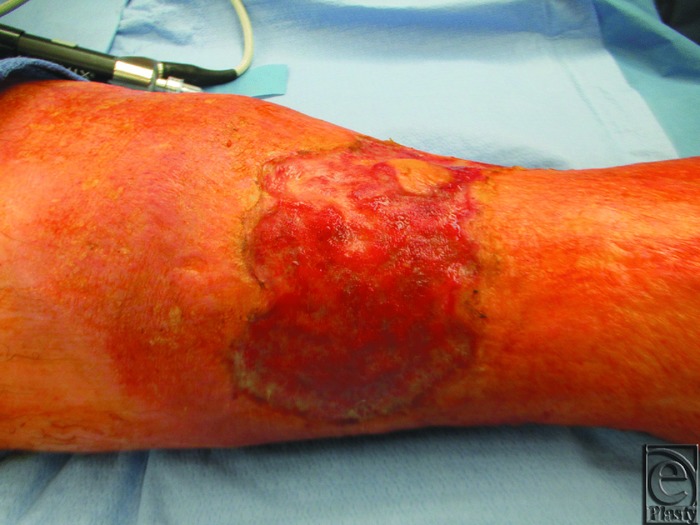

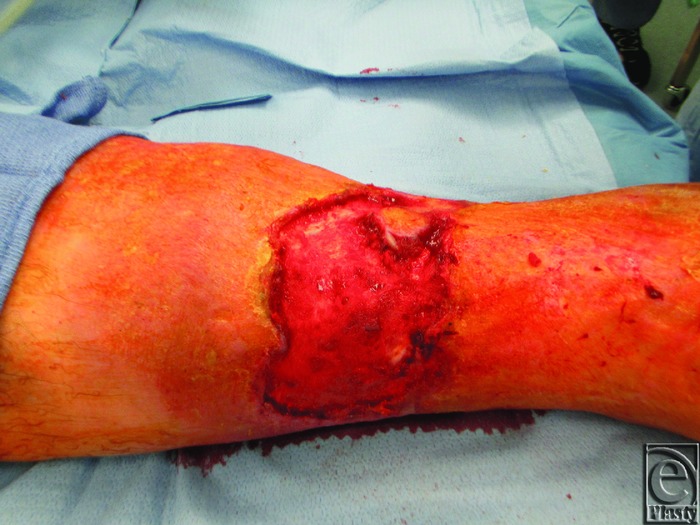
Outpatient Debridement with Low Frequency Contact Ultrasound This was a prospective, randomized, controlled, clinical study involving 76 subjects with venous ulcers over a 24-week period in an outpatient hospital-based wound care center. The ultrasonic debridement procedures were performed using a low-frequency (22.5 kHz), high-intensity (∼60 W/cm^2^) contact ultrasound device that produces cavitation. Wounds debrided ultrasonically healed significantly faster than wounds debrided with sharp debridement (curettage). The incidence of complete healing was significantly greater (*P* < .05) after 20 and 24 weeks in subjects receiving ultrasonic debridement.

Debridement of nonviable tissue is necessary for wound improvement and healing and is considered a cornerstone of acute or chronic wound management. Research has shown that removing nonviable tissue decreases the incidence of infection and accelerates the rate of closure.^1^^,^^2^ Direct-contact, low-frequency ultrasound (DCLFU) combines the effectiveness of sharp debridement with the benefits of ultrasound therapy.^3^^-^6 A recent systematic review of the literature and meta-analysis of low-frequency ultrasound as adjunctive therapy for chronic wound healing concluded that both low-frequency, low-intensity ultrasound and low-frequency, high-intensity ultrasound were beneficial to healing.^3^ The benefit of the low-frequency, high-intensity ultrasound method is that it incorporates a probe to excise nonviable tissues and facilitate wound debridement.^4^ DCLFU also produces an additional acoustic phenomenon called bubble cavitation. Bubble cavitation is the creation and subsequent collapse (destruction) of small bubbles within the fluid surrounding the probe releasing mechanical energy.^7^ During cavitation, the bubbles oscillate in size and shape. The oscillation of the bubbles is dependent on the frequency of the ultrasound wave. The bubbles expand and rapidly collapse, causing acoustic streaming.^8^ DCLFU provides both mechanical and hydrodynamic effects directly in the wound bed. This method causes necrotic tissue disruption, fragmentation, and emulsion.^7^^,^^9^

## STUDY OBJECTIVE AND ENDPOINTS

The objective of this study was to evaluate and compare the effects of 2 different debridement methods (surgical sharp debridement and debridement with DCLFU) on the healing of chronic venous ulcers. The primary endpoint was time to complete wound healing, and secondary endpoints were relative rate of wound healing and total number of debridement procedures per patient episode.

## METHODS

Each subject with a venous ulcer requiring debridement was randomized to receive either ultrasonic debridement (*N* = 36) or sharp debridement with a curette (*N* = 40).

Study inclusion criteria were as follows: the patient's wound had to be of venous etiology, the wound needed debridement, the target ulcer's surface area had to be more than 3 cm^2^, the ulcer's history of nonhealing had to be more than 4 months, and the patient's affected leg had adequate arterial blood flow (ankle-brachial index [ABI] >0.7). The exclusion criteria were as follows: if the patient had a bleeding disorder, an ABI of less than 0.7, uncontrolled diabetes, taking systemic corticosteroids, had chemotherapy or radiotherapy, participating in another study, and had been treated with Apligraft, Dermagraft, or Regranex within 90 days.

**Figure 1:**
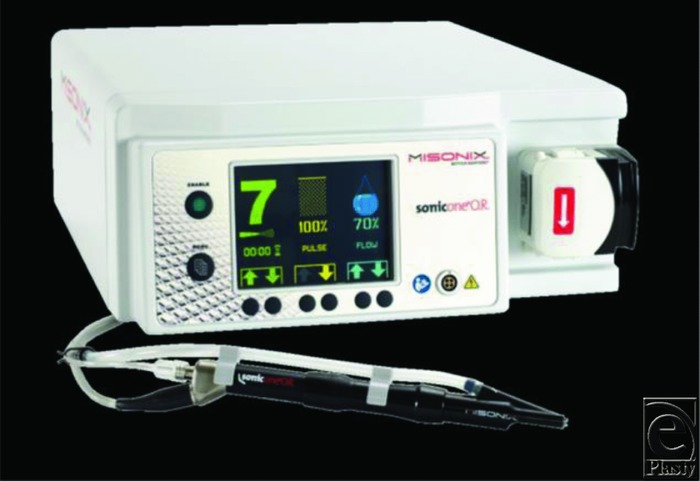
SonicOne ultrasonic debridement system combines aspiration with ultrasonic debridement.

The device used for DCLFU (Fig 1) was SonicOne (Misonix Inc, Farmingdale, NY).

Utility-supplied electrical energy is converted by the SonicOne ultrasonic generator to a 22.5-kHz high-frequency signal. The amplitude of this signal is user adjustable by an amplitude control dial on the generator itself. The electrical signal is then converted to vibratory energy by the transducer (in the handpiece), which contains a piezoelectric stack (literal meaning: pressure from electricity). The vibrations of the transducer's distal surface are amplified by means of a titanium probe that is attached to the transducer by a screw thread. The probe not only amplifies the motion of the transducer face but also provides a means to project the vibrating surface deeper into the tissue bed. The selected probe also tailors the energy input to ablate or emulsify the unwanted nonviable tissue types. The operating tips can also be easily changed from patient to patient to prevent cross contamination and maintain sterility. The vibrations (energy) that are transmitted longitudinally along the length of the probe are brought into direct contact with the tissue at the wound site. The necrotic tissue, eschar, slough, or fibrin is cut/excised, dissected, fragmented, or ablated—depending upon the probe design and the level of vibratory energy transmitted down the probe. The varieties of probes that are used are designed to concentrate (sharper tips) or disperse (blunter tips) the vibratory energy, resulting either in differing aggressiveness of the dissection or fragmentation of the soft tissues. Depending upon the probe design and the level of vibratory energy transmitted, cavitational effects can occur at the tissue surface and within the tissue itself. The ultrasonic energy may be kept constant or “pulsed” into the wound. It has been found that by pulsing the input energy, patient pain and discomfort are diminished.^10^ The major components of the ultrasonic debridement device with the different probes are shown in the Figure 1.

Standard sharp debridement could be performed with a curette, scalpel, or scissors. For both, ultrasonic and sharp debridement interventions, local anesthesia was achieved with topical lidocaine 4% gel kept under occlusion with a polyurethane film wound dressing (Tegaderm; 3M, St Paul, Minn). The study subjects were treated with the topical lidocaine 15 to 20 minutes prior to the procedure. Hemostasis (in both intervention groups) was achieved with compression alone or ferric subsulfate (Monsel's solution) plus compression. Debridement with either intervention could be performed (as needed) at the investigator's discretion. Patients in both interventional groups were treated with the same primary dressing and the same compression therapy. The primary dressing was Mepilex Wound Dressing (Molnlycke USA, Norwood, Ga) and compression therapy was with a multilayer compression bandage system (Profore; Smith and Nephew, Inc, Largo, Fla). Measurements of the wound and percentage of nonviable and granulation tissues were made from digital photographs^11^ using a photodigital wound measurement software program (Pictzar, BioVisual Inc, Elmwood Park, NJ).

Statistical analysis was based on intent to treat. Fisher's exact test was used to test for differences in the number of debridement procedures and differences in wound pain. The Kaplan-Meier survival analysis was used to measure time to healing, and healing rate was measured using the log-rank χ^2^ test. Time in motion and number of procedures were compared with Student's *t* test.

## RESULTS

**Figure 2:**
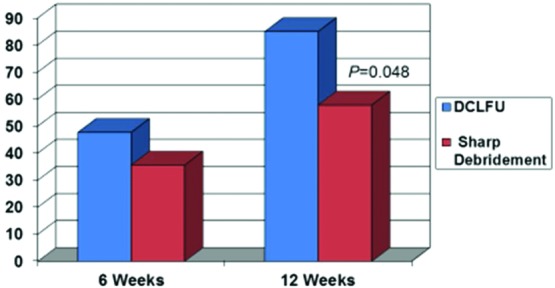
Mean percent reduction in wound surface area at 6 and 12 weeks.

**Figure 3:**
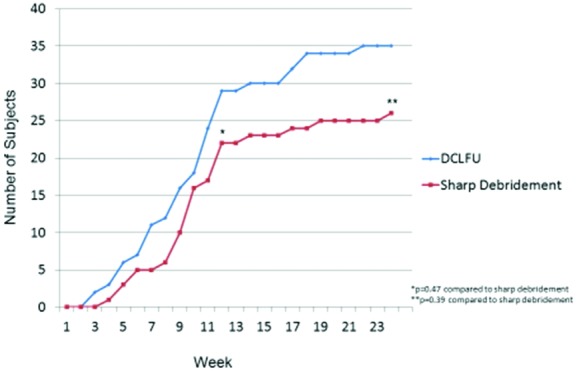
Incidence of complete healing in subjects receiving ultrasonic or sharp debridement.

Patient demographics between the 2 intervention groups are presented in Table 1. There were no statistically significant differences between the 2 groups. The frequency of debridement procedures in each intervention group is presented in Table 2. Subjects receiving ultrasonic debridement required 40% fewer procedures than those receiving sharp debridement (*P* < .05). The healing rate of ulcers is presented in Figure 2. The rate of wound closure in the ultrasonic debridement group was 25% and 32% greater at 6 and 12 weeks, respectively. At the 12-week time point, the difference in the rate of healing was statistically significant (*P* = .048) (Fig 3).

The greater effectiveness of ultrasonic debridement over sharp debridement was also evident on survival analysis by the Kaplan-Meier life table method at 24 weeks (Fig 3).

Seventy-three percent of the subjects in the ultrasonic debridement group healed compared with 54% in the standard sharp debridement group (*P* = .044).

**Figure 4:**
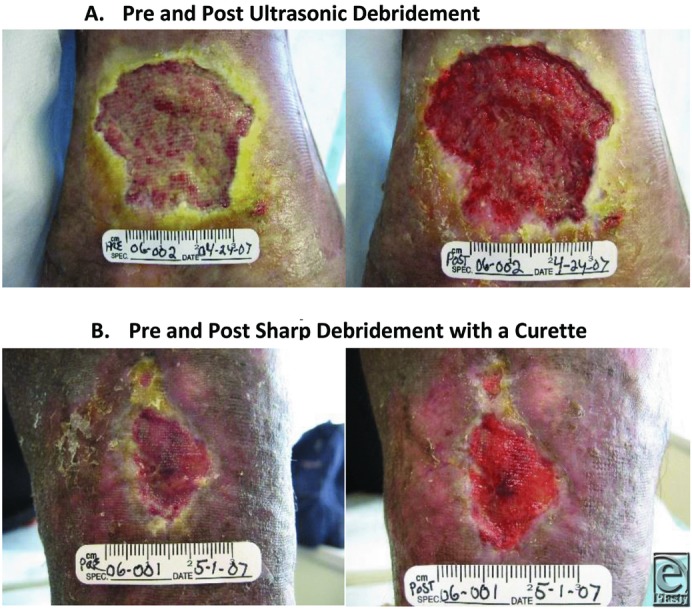
(*a*) Pre- and post–ultrasonic debridement. (*b*) Pre- and post–sharp debridement with a curette.

Photographs of before and after debridement procedures are shown in Figure 4.

## CONCLUSIONS

Wounds debrided with contact ultrasound healed significantly faster than wounds debrided with sharp debridement. The incidence of complete healing was significantly greater (*P* < .05) after 20 and 24 weeks in subjects receiving ultrasonic debridement. Debridement with contact ultrasound resulted in significantly fewer procedures and faster healing and focuses on the value of patient-centered care (improving clinical outcome while reducing costs).

## Figures and Tables

**Table 1:** Patient demographics between the 2 intervention groups*

	Intervention	
Variable	Ultrasonic debridement	Standard sharp debridement
Subjects	36	40
Mean age, y	64	60
Sex (% female)	42	51
Ulcer age, mo	8	4
Baseline ulcer area, cm^2^	11.23	9.89
Pain intensity at baseline (mean VAS)	3.91	4.25

*VAS indicates visual analog scale.

**Table 2:** Total number of debridement procedures and frequency per subject

	Total number of	Number of debridement procedures/subject,
Procedure	debridement procedures	mean ± SEM
Ultrasonic debridement	126	3.5 ± 2.9
Sharp debridement	232	5.8 ± 5.2
